# 

**Published:** 1890-12

**Authors:** 


Died.—The JOURNAL regrets to learn of the death of Dr. J. H. Smith, late Oculist of Dallas. He died in Brooklyn, recently, where he had gone on a visit. Dr. Smith was a member of the Texas State Medical Association.

